# Correlation between intelligence, level of formal education, and nerve transfers for elbow flexion recovery outcomes following brachial plexus reconstructive surgery

**DOI:** 10.1016/j.bas.2026.105983

**Published:** 2026-03-17

**Authors:** Simon Miedema, Ana Carolina Lovaglio Rivas, Martin Bourguet, Maria Roca, Gilda Di Masi, Gonzalo Bonilla, Brenda Iglesias, Danilo Bataglia, Mariano Socolovsky

**Affiliations:** a Division of Neurosurgery, Leiden University Medical Center, Leiden, the Netherlands; b Peripheral Nerve and Brachial Plexus Surgery Department, Division of Neurosurgery, Hospital de Clínicas “José de San Martín”, University of Buenos Aires, Buenos Aires, Argentina; c Cognitive and Translational Neurosciences Institute (INCyT)-INECO-Favaloro University-CONICET, Buenos Aires, Argentina

**Keywords:** Brachial plexus injury, Intelligence, Elbow flexion recovery

## Abstract

**Background:**

Several factors have been shown to influence post-surgical recovery for elbow flexion repair after nerve transfers for brachial plexus surgery. The brain's impact on surgical outcomes has garnered considerable attention, prompting an investigation into the influence of various cortical functions, including intelligence and academic achievement. The aim of this study is to investigate whether intelligence is correlated with functional outcomes of elbow flexion following nerve transfer.

**Methods:**

We conducted a retrospective study of patients who underwent nerve transfers to restore elbow flexion. Intelligence was assessed using the WAT BA and Matrix Reasoning subtest (MRT) of the WAIS-III, and the grade of scholarship was evaluated based on the years and levels of education completed. Motor recovery was assessed through the British Medical Research Council (BRMC) scale and strength index scores.

**Results:**

A total of 71 patients were included, with a mean Intelligence score of 89 (range 67–114). Intelligence correlated with BRMC motor outcomes (p = 0.023, r = 0.270). Additionally, patients with lower intelligence scores (<85) experienced a prolonged time from trauma to surgery (p = 0.017) and lower rehabilitation quality scores (p = 0.001, CC = 0.398). Notably, patients with ≤7 years of education and lower scholarship levels demonstrated significantly lower BRMC scores (p = 0.049).

**Conclusion:**

A positive association was found between higher Intelligence, and motor recovery following nerve transfer surgery. This relationship may be attributed to direct differences in cortical abilities and to indirect factors, such as delayed surgery and suboptimal adherence to rehabilitation.

## 1 Background

Traumatic brachial plexus injuries are a frequent cause of permanent disability in young, economically active individuals (Faglioni et al., 2014; Flores, 2006; Jaquet et al., 2001). Modern microsurgical techniques have improved outcomes in the treatment of these lesions, with nerve transfers playing a leading role. Timely intervention, a young age, a lower body mass index (BMI), and adequate physiotherapy, among other factors, have been identified as predictors of favorable outcomes (Socolovsky et al., 2011, 2014; Socolovsky et al., 2011; Bhatia et al., 2020; Flores, 2006; Yoshikawa et al., 2012). Identifying other variables that positively impact the results of reconstructive surgery is critical for selecting appropriate surgical candidates, refining therapeutic planning, and enhancing rehabilitation efforts (Socolovsky et al., 2017).

Brain changes occur immediately after injury, leading to cortical deafferentation (Yoshikawa et al., 2012; Socolovsky et al., 2017). Nerve transfers and postoperative rehabilitation modify the original cortical motor programs, allowing previously denervated regions to gradually regain motor control and sensory perception (Yoshikawa et al., 2012; Socolovsky et al., 2017; Anastakis et al., 2008). This process leads to the remodeling of the cortical map, impacting postoperative functionality (Shen, 2022; Li et al., 2021; Kleim, 2011; Fujiwara et al., 2019). The increasing recognition of the role of brain changes in the context of nerve transfers after brachial plexus reconstructive surgery has prompted further inquiry into how overall brain function may influence recovery outcomes, potentially including intellectual ability, determined by the intelligence as an influential variable affecting the results of nerve transfers (Brans et al., 2010). Prior research has demonstrated that reduced cortical thickness is associated with a lower IQ, indicating a relationship between brain structure and cognitive function (Burgaleta et al., 2014; Bajaj et al., 2018). Moreover, IQ significantly influences structural differentiation of the brain during adolescence (Koenis et al., 2018), and muscle strength has been correlated with short-term memory performance (Agostino et al., 2023). These findings prompt the question of whether intelligence level, characterized by greater learning capacity and increased neuronal connections, may act as an additional factor influencing motor recovery in patients following surgery.

This study aims to investigate the relationship between fluid intelligence, defined as the capacity to think and reason flexibly when solving new and abstract problems, the level of formal education, and motor recovery, specifically focusing on elbow flexion, following reconstructive surgery in patients with brachial plexus injuries.

## 2 Methods

### 2.1 Study design

A retrospective study was conducted involving patients who underwent a nerve transfer procedure aimed at restoring elbow flexion as part of brachial plexus reconstruction. The inclusion criteria mandated that participants had: (1) received a nerve transfer specifically for elbow flexion, (2) completed a minimum follow-up period of one year, with the exception of patients who achieved a British Medical Research Council (BMRC) grade 4 strength within the first year, and (3) provided informed consent. All surgeries were performed at the Division of Neurosurgery at the Hospital de Clínicas “José de San Martín" between April 2007 and October 2023.

Demographic and therapeutic data were collected, including the patient's age at the time of surgery, sex, interval between trauma and surgery, type of nerve lesion, surgical procedure performed, years of education, and the duration and quality of postoperative rehabilitation. The strength of elbow flexion after the final follow-up was determined using both the BRMC scale and dynamometer readings, quantified in kilograms, and compared with measures obtained from the healthy arm. This comparison yielded an index that divided the unaffected side by the affected side, which can range from a maximum value of 1 to a minimum of 0. Additionally, brain plasticity was assessed using the established four-point Plasticity Grading Scale, as previously described (Socolovsky et al., 2022) (Table 1). This assessment was exclusively performed in patients with a British Medical Research Council (BMRC) score of 4, as meeting this criterion is a prerequisite for applying the grading scale. Rehabilitation compliance was assessed utilizing the 4-point Rehabilitation Quality Scale (RQS). Patients who did not attend therapy or attended less than once a week were assigned an RQS score of 1. A score of 2 was assigned to those who regularly attended rehabilitation more than once a week at a non-specialized center. A score of 3 was assigned for good adherence at a non-specialized center with periodic evaluations at a specialized neurorehabilitation facility. Patients with strong adherence at a specialized neurorehabilitation center were given a score of 4. All included patients demonstrated adequate understanding of the surgical procedure and rehabilitation protocol. Preoperatively, patients were instructed to practice the target movements using the unaffected limb to facilitate motor learning and prevent joint stiffness.

**Table 1:** Plasticity Grading Scale for nerve transfers.

Grade	Target Muscle Contraction ↔ Motor Program Activation	MRC Grade of Target Muscle Contraction	
		Donor Command	Acceptor Command
1	Exclusively donor (no plasticity)	4	0
2	Via donor & acceptor (poor plasticity)	2, 3, or 4	4
3	Subtle via donor, predominantly via acceptor (good plasticity)	1	4
4	Exclusively acceptor (excellent plasticity)	0	4

MRC: Medical Research Council Scale.

### 2.2 Intelligence measurements

Premorbid intelligence was evaluated with the WAT BA, while fluid intelligence (FI) was measured using the Matrix Reasoning subtest (MRT) of the WAIS-III, which was administered by trained neuropsychologists who were not involved in the patients' treatment.

The WAT BA Test involves reading a series of standardized words without accentuation. The patient will accentuate the words while reading, and responses will be recorded against a control sheet of correct answers. The number of correct answers will be documented as the raw score of the WAT BA Test. Correct accentuation reflects the patient's retained knowledge and cognitive abilities before potential cognitive decline (premorbid intelligence) (Sierra et al., 2010). The Matrix Reasoning subtest (MRT) of the WAIS-III, the available version at the time this study was launched, is designed to measure nonverbal reasoning and problem-solving skills. Patients are required to identify the missing piece in a series of visual patterns, providing insight into their fluid intelligence and abstract thinking capabilities (Wechsler, 2002). The raw scores of both tests were adjusted for age and converted into equivalent scores of fluid intelligence, according to standardized tables (Del Ser et al., 1997; Sierra et al., 2010; Wechsler, 2002).

The total years of formal education completed were recorded for each individual and categorized to specific education levels. Individuals were classified as having elementary education if they completed up to 7 years of formal education, secondary education for 8 to 12 years, and high school or tertiary/university education for more than 13 years.

### 2.3 Surgical procedures

The surgical procedures involved either supraclavicular, infraclavicular, or combined approaches to perform nerve transfers. For elbow flexion, reinnervation was directed to the musculocutaneous nerve or its branches supplying the biceps or brachialis muscles. Potential donor nerves included the spinal accessory nerve, phrenic nerve, intercostal nerves, flexor carpi branches of the ulnar or median nerves, and preserved C5 spinal root. In some instances, nerve grafts were employed to prevent tension at the suture sites. All patients underwent nerve reconstruction within one year post-trauma, except Oberlin procedures.

### 2.4 Statistical analysis

A scaled score of 7 or lower in the WAIS Matrices subtest indicated low performance in fluid reasoning or fluid intelligence (FI), corresponding to a standard intelligence score of 85. This criterion allowed for the identification of subjects whose performance fell one standard deviation below the mean. Using a similar methodology, scores between 85 and 114 were classified as having moderate intelligence, while scores of 115 and above were categorized as high.

All outcomes were tested for normality. Since none of the groups exhibited a normal distribution, the Mann-Whitney and Chi-square tests were used to evaluate the differences between groups. Additionally, the impact of age, lesion type, time from trauma to surgery, type of surgery, and the interval from surgery to FI test assessment on elbow flexion function was examined using Chi-square and Kruskal-Wallis tests. Continuous data are presented as median and percentiles [25th:75th].

Spearman's rank correlation was employed to assess the relationships between cognitive scores, measured by WAT BA and Matrices tests, categorized years of education, surgical timing, RQS scores, PGS outcomes, and biceps function as assessed by the BRMC scores and the elbow flexion index described above. The association between categorized years of education and BMRC outcomes was additionally evaluated using the Chi-square test. All statistical tests were performed two-sided with a significance level set at alpha 0.05.

## 3 Results

A total of 71 patients who met the inclusion criteria were analyzed, with a mean age of 28 years [24:36]; of these, 67 (94%) were male. The median time from trauma to surgery was 6 months [4:9], and the median follow-up duration was 36 months [20:56]. The distribution of brachial plexus injury types was as follows: C5-T1 in 44 patients (62%), C5-C6 in 16 patients (23%), C5-C7 in 7 patients (10%), C5-C8 in 3 patients (4%), and C6-T1 in 1 patient (1%).

The median intelligence score for the population was 90 [79:98], with a range of 67 to 114. The characteristics of the population are described in Table 2. A total of 24 patients were classified as having a low FI level (below 85), 47 patients as having a moderate FI (85–114), and no patients were classified into the high FI category (≥115).

**Table 2:** Comparison of clinical and demographic factors between patients with Low (<85) and Moderate (85–114) intelligence scores measured as Fluid Intelligence (FI).

Variable	Total n = 71	Low FI n = 24	Moderate FI n = 47	P-value∗
Age at time of surgery, years	28 [24:36]	28 [23:36]	29 [25:37]	0.531
Sex, male (%)	94	100	91	0.141
Time from trauma to surgery, months	6 [4:9]	8 [5:10]	5 [3:7]	0.017
Duration of follow-up, months	36 [20:56]	33 [14:49]	37 [26:58]	0.114
Type of lesion (%)				0.674
C5-C6	16	5	11	
C5-C7	7	3	4	
C5-C8	3	2	1	
C6-T1	1	0	1	
C5-T1	44	14	30	
Type of surgery (%)				0.728
Single ulnar or median	8	3	5	
Double ulnar and median	16	5	11	
XI-AD	4	1	3	
XI-MC	10	6	4	
C5-AD	1	0	1	
C5-MC	2	1	1	
Fr-AD	16	5	11	
Fr-MC	13	3	10	
RQS (n = 69)	3 [2:4]	3 [2:4]	3 [3:4]	0.174
PGS (n = 40)	3 [2:3]	3 [2:3]	3 [2:3]	0.634

(XI-Da = Accessory nerve to the anterior division of the upper trunk; XI-MC = Accessory nerve to the musculocutaneous nerve; C5-AD = Cervical nerve 5 to the anterior division of the upper trunk; C5-MC = Cervical nerve 5 to the musculocutaneous nerve; Fr-AD = Phrenic nerve to the anterior division of the upper trunk; Fr-MC = Phrenic nerve to the musculocutaneous nerve, FI = Fluid Intelligence. ∗ = analysis conducted between low and moderate FI groups).

A significant positive correlation was observed between WAT BA test scores, MRT scores, and BRMC outcomes (p = 0.023, r = 0.270; p = 0.031, r = 0.256, respectively), indicating that higher cognitive scores were linked to improved motor recovery. This correlation remained statistically significant after multivariable adjustment for age, type of injury, and type of nerve surgery (WAT-BA: p = 0.015, r = 0.352; MRT: p = 0.038, r = 0.304).

Additionally, years of education were positively correlated with BRMC outcomes (p = 0.004, CC = 0.338), as well as with intelligence, assessed by MRT (p < 0.001, CC = 0.574) and WAT BA (p < 0.001, CC = 0.857). However, no significant association was observed between years of education and PGS outcomes (p = 0.693, CC = −0.064).

**Fig. 1:**
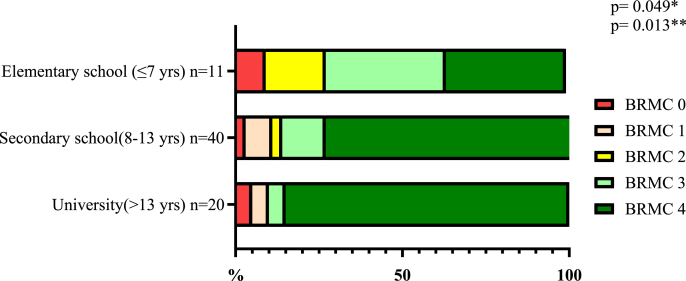
Association between years of education and BRMC outcomes (*∗ = Analysis of BRMC grades 0-1-2-3-4, ∗∗ = Comparison of BRMC grades 0-3 versus 4 across all subgroups*).

When years of education were categorized into distinct levels—primary school (up to 7 years of education), secondary school (8-13 years), and higher education (more than 13 years)—a significant association with BRMC outcomes emerged (p = 0.049, Chi-square test), as shown in Table 3 and Fig. 1. Specifically, among patients with primary school education, only 36% (4/11) achieved a BRMC score of 4. In contrast, this rate increased to 75% (30/40) among those with secondary education and further to 85% (17/20) for patients with higher education.

**Table 3:** Association between years of education and BRMC outcomes.

n (%)	BRMC 0	BRMC 1	BRMC 2	BRMC 3	BRMC 4	P-value
Primary school (≤7 yrs)	1 (9)	0 (−)	2 (18)	4 (36)	4 (36)	0.049∗ 0.013∗∗
Secondary school (8-13 yrs)	1 (2.5)	3 (7.5)	1 (2.5)	5 (12.5)	30 (75)	
Higher education (>13)	1 (5)	1 (5)	0 (−)	1 (5)	17 (85)	

(∗ = Analysis of BRMC grades 0-1-2-3-4, ∗∗ = Comparison of BRMC grades 0-3 versus 4 across all subgroups).

The interval between trauma and surgery varied significantly among groups differentiated by cognitive ability (p = 0.017). Those classified as having low FI experienced a longer interval, averaging 8 months [5:10], compared to 5 months [3:7] for those with moderate FI (Table 2). Additionally, the time from trauma to surgery showed a negative correlation with the strength index of the biceps muscle (p = 0.009, r = −0.326).

Lastly, a significant positive correlation was observed between RQS and MRT as well as years of education (p = 0.001, CC = 0.398 and p = 0.013, CC = 0.311, respectively), indicating that patients with higher fluid intelligence scores tended to achieve better rehabilitation quality outcomes.

No effect was observed between the time from trauma to surgery, age at time of surgery, duration of follow-up, type of lesion, and nerve transfer on BRMC scores. Similarly, no correlation was identified between PGS scores or the extent of traumatic brain injury and FI, premorbid intelligence measurements.

## 4 Discussion

The observed relationship between higher intelligence scores and more years of formal education, both of which correlate with better outcomes for elbow flexion restoration after surgery, is the main original finding of this series. It can be understood through three mechanisms. First, cortical abilities play an important role. Individuals with a higher FI tend to have greater cognitive flexibility and adaptability (Garlick, 2002; Brehmer et al., 2014), which may enhance their capacity to recover from nerve surgery by facilitating cortical changes (Rosén et al., 1994). This concept is commonly referred to as “cognitive reserve,” a mechanism that enables individuals to achieve greater neural efficiency, expanded neural capacities, and the ability to compensate by recruiting additional brain regions (Tucker and Stern, 2011). Thus, possessing more cognitive compensatory mechanisms may enhance overall motor recovery and improve functional outcomes following surgical interventions. Secondly, the time interval between trauma and surgery was longer for patients with lower intelligence scores. Previous studies have shown that extended delays before surgery are associated with poorer outcomes (Bhatia et al., 2020), suggesting that this may mediate the assessment of the impact of intelligence on elbow flexion function. The longer interval may be due to reduced awareness or understanding of the need for timely medical intervention, as well as other factors such as limited access to care. Of note, in cases of severe polytrauma, the diagnosis of peripheral nerve injury is frequently delayed due to prolonged intensive care unit admission and the prioritization of life-threatening injuries, which substantially extend the time to referral and surgical intervention. Lastly, lower FI scores have been linked to decreased engagement in rehabilitation efforts (Dutzi et al., 2021), which is crucial for the recovery of elbow flexion (Novak and von der Heyde, 2015; Maugeri et al., 2021). Patients with lower intelligence may face financial constraints or lack motivation to fully commit to rehabilitation programs (Verrienti et al., 2023; Ganuthula and Sinha, 2019), which are vital for optimizing post-surgical outcomes. Thus, the influence of intelligence on recovery could be attributed to a combination of these interconnected variables.

Neuroplasticity, the capacity of the central nervous system to reorganize and adapt in response to internal or environmental changes (Mateos-Aparicio and Rodríguez-Moreno, 2019), plays a crucial role in optimizing functional outcomes (Socolovsky et al., 2017; Anastakis et al., 2008). This dynamic process involves the adaptation and modification of neuronal connections in response to stimuli (Socolovsky et al., 2017; Anastakis et al., 2008; Garlick, 2002; Nelson, 1999). In recent years, significant advances have been made in understanding brain development and information processing (Garlick, 2002). It is believed that neural flexibility is critical for the proper processing of information (Kuhl et al., 2020), with activation patterns within neural networks being shaped and modified by changes in synaptic connections. In newborns, these neural connection networks are relatively undifferentiated, but they progressively become more intricate throughout childhood and into adulthood (Koenis et al., 2018; Hochberg and Konner, 2020; Arain et al., 2013). Within the context of peripheral nerve injury repair, existing literature has demonstrated a correlation between cognitive flexibility, intelligence levels, and sensory recovery in the hands (MahmoudAliloo et al., 2011). Moreover, networks with higher learning capacities and adaptable connections, such as those characterized by neuroplasticity, have demonstrated better performance on certain intelligence tests (Garlick, 2003).

Given the retrospective nature of the study, we were unable to investigate the precise causal mechanism underlying the direct, positive relationship between years or levels of education and the BRMC outcomes observed herein. Following the discovery of a correlation between intelligence scores and previously analyzed elbow flexion recovery results, this was the second most important original finding in our series. One potential explanation for that relationship is the “use-it-or-lose-it” principle of brain plasticity, where greater complexity in one's primary occupation is associated with enhanced cognitive performance (Lövdén et al., 2013). Following brain injuries, individuals with higher intelligence and advanced education demonstrate less impairment compared to those with lower intelligence and limited educational backgrounds (Wilson, 2010). In addition, a previous study has shown that individuals who maintain mental activity and engage in cognitively demanding tasks exhibit greater brain plasticity than those who have retired (Finkel et al., 2009). Alternatively, this relationship may be primarily driven by cognitive capacity, as individuals with higher cognitive abilities tend to have more years of education and demonstrate superior outcomes.

The concept of intelligence has been a subject of research for centuries, evolving from a single “general intelligence” factor, known as factor g (Ardila, 2023; Anderson, 1995), to more nuanced theories of multiple intelligences and a combination of factors (Shearer, 2018; Thomas, 2016). In our study, the MRT was employed to measure fluid intelligence, a test that heavily relies on the foundational concept of factor g (Colom et al., 2002; Ardila, 2023; Roca et al., 2010). Moreover, the WAT BA is commonly used as an indicator of cognitive function prior to cognitive decline, such as in cases of dementia, raising questions about its suitability for our analysis.

A key limitation of this study lies in the relatively small sample size, with a large portion of patients exhibiting high BMRC grades, notably 72% scored grade 4. This likely reduced the statistical power necessary to conclusively determine which specific intelligence score groups were associated with poorer outcomes. Additionally, the absence of patients with high FI scores (>115) further limited our ability to explore the relationship between cognitive ability and functional recovery. Consequently, it was not possible to determine whether individuals with high intelligence levels exhibit superior outcomes following nerve surgery or if a plateau effect exists beyond moderate intelligence scores. The heterogeneity of the patient population further restricted the ability to evaluate the impact of intelligence on functional recovery across various types of lesions. In addition, socioeconomic factors and access to care influence both educational attainment and treatment timelines; however, these variables were not directly captured in the present study and should be addressed in future investigations. To overcome these limitations, future research should prioritize multicenter prognostic studies in more homogeneous patient cohorts, combined with standardized, high-adherence rehabilitation protocols, to more precisely isolate the independent contribution of cognitive factors to functional recovery.

In conjunction with rehabilitation, nerve reinnervation typically yields favorable outcomes for elbow flexion recovery (Vyas et al., 2024; Venkatramani et al., 2008; Lee et al., 2024). However, several factors are known to impact post-surgical results (Chuang et al., 1993). These include the level of the lesion (proximal or distal), the presence of nerve root avulsion, the number of axons reaching the target muscle, and the extent of the lesion (Socolovsky et al., 2014; Neti et al., 2022). Additionally, our findings suggest that intelligence is another variable that influences recovery, likely both directly through cortical abilities and indirectly through its association with more extended time intervals between trauma and surgery, as well as engagement in rehabilitation. When informing patients about their prognosis and selection for additional rehabilitation programs, these factors should be carefully considered. Importantly, IQ should not be interpreted as a determinant of eligibility or expected benefit, but rather as a consideration for tailoring rehabilitation strategies to the patient's cognitive profile. Recognizing cognitive diversity allows surgical and rehabilitation programs to be adapted in content, intensity, and delivery, thereby reducing the risk of stigmatization while ensuring equitable access to effective postoperative care. Such an approach emphasizes equity rather than exclusion and supports optimal functional recovery across the full spectrum of intellectual abilities.

## 5 Conclusion

Our findings indicate that patients with lower cognitive scores and lower levels of formal education tend to experience poorer motor recovery outcomes. This association may be influenced by factors such as the delayed time from trauma to surgery and the quality and adherence of rehabilitation. To further elucidate this relationship and identify modifiable factors that could enhance motor recovery following peripheral nerve surgery, larger multicenter studies involving diverse patient populations and surgical teams are required.**References**.

## Declaration of competing interest

The authors declare the following financial interests/personal relationships which may be considered as potential competing interests:Simon Miedema reports was provided by Leiden University Medical Center. Nothing to declare If there are other authors, they declare that they have no known competing financial interests or personal relationships that could have appeared to influence the work reported in this paper.
